# A Peptidomimetic Fluorescent Probe to Detect the Trypsin β2 Subunit of the Human 20S Proteasome

**DOI:** 10.3390/ijms21072396

**Published:** 2020-03-31

**Authors:** Magdalena Wysocka, Anita Romanowska, Natalia Gruba, Michalina Michalska, Artur Giełdoń, Adam Lesner

**Affiliations:** Faculty of Chemistry, University of Gdansk, Ul. Wita Stwosza 63, 80-308 Gdansk, Poland; magdalena.wysocka@ug.edu.pl (M.W.); anita_romanowska@o2.pl (A.R.); natalia.gruba@ug.edu.pl (N.G.); michalinamichalska@icloud.com (M.M.); artur.gieldon@ug.edu.pl (A.G.)

**Keywords:** peptidomimetics, libraries, fluorogenic substrates, proteasome, bladder cancer

## Abstract

This work describes the chemical synthesis, combinatorial selection, and enzymatic evaluation of peptidomimetic fluorescent substrates specific for the trypsin-like (β2) subunit of the 20S human proteasome. After deconvolution of a library comprising nearly 6000 compounds composed of peg substituted diaminopropionic acid DAPEG building blocks, the sequence ABZ–Dap(O2(Cbz))–Dap(GO1)–Dap(O2(Cbz))–Arg–ANB–NH_2_, where ABZ is 2-aminobenzoic acid, and ANB- 5 amino 2- nitro benzoic acid was selected. Its cleavage followed sigmoidal kinetics, characteristic for allosteric enzymes, with *K*_m_ = 3.22 ± 0.02 μM, *k*_cat_ = 245 s^−1^, and *k*_cat_/*K*_m_ = 7.61 × 10^7^ M^−1^ s^−1^. This process was practically halted when a selective inhibitor of the β2 subunit of the 20S human proteasome was supplemented to the reaction system. Titration of the substrate resulting in decreased amounts of proteasome 20S produced a linear signal up to 10^−11^ M. Using this substrate, we detected human proteasome 20S in human urine samples taken from the bladders of cancer patients. This observation could be useful for the noninvasive diagnosis of this severe disease.

## 1. Introduction

The 20S proteasome has a tube-like structure composed of four rings formed by 28 protein subunits [1,2]. The external rings are composed of seven distinct α subunits while the internal rings are composed of seven diverse β subunits. All α subunits have structural functions, whereas only three of the β subunits (β1, β2, and β5) have active centers (catalytic sites) [3]. In each of these β subunits, a threonine (Thr) residue is positioned at the N-terminal. This amino acid is crucial for the proteolytic activity of the enzyme.

The cleavage specificity of each catalytically active β subunit is defined by the structure of its S1 binding pocket, particularly the chemical properties of the amino acid residue located at position 45 of the proteasome structure [4]. The β1 subunit contains Arg at this position in the vicinity of the binding cleft, thereby accommodating and recognizing the acidic amino acids Glu and Asp and cleaving the peptide bond at the C-terminal side. At position 45 of the β2 subunit, Gly cooperates with its neighbor Glu53, and accepts positively charged amino acid residues such as Lys and Arg. This assembly is referred to as trypsin-like activity. Lastly, the β5 subunit, with Met45 in the substrate pocket [5,6], cleaves peptide bonds on the carboxyl side of hydrophobic amino acids (aliphatic and aromatic).

Elevated 20S proteasome activity has been reported in several human diseases, including lupus erythematosus [7], rheumatoid arthritis [8], and various kinds of malignancies. For example, catalytic overexpression of the 20S proteasome core has a diagnostic value in disease therapy [9]. The origin of its presence outside of the cell remains unclear, with two different hypotheses having been proposed. The first suggests that the presence of the 20S proteasome is an effect of cell death by necrotic- or macrophage-mediated cytolysis. The second postulates that the 20S proteasome is related to the abnormally fast division of cancer cells [7]. Proteasome 20S secreted outside the cell has the most significant diagnostic value and this form is referred to as the circulating proteasome. Its presence and associated increased enzymatic activity has been observed in several kinds of cancer, including breast tumors [10], prostate cancer [11], head and neck neoplasms [12], and ovarian cancer [7,13].

Common examples are patients with multiple myeloma, whose circular 20S proteasome concentrations range between 1.81 and 11.06 μg/mL. However, for healthy people, this value never exceeds 1 μg/mL [14,15]; thus, to facilitate the development of a simple and robust proteasome assay, novel and sensitive tools to study proteasome activity are required.

**Figure 1:**
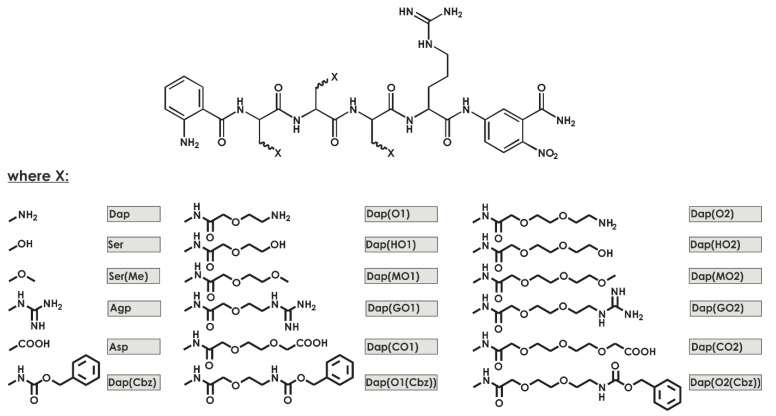
General formula for the library and its components. L-2,3-diaminopropionic acid (Dap), Ser, Ser(Me), L-2-amino-3-guanidino-propionic acid (Agp), Asp, Dap(Cbz), 5-(t-butyloxycarbonyl-amino)-3-oxapentanoic acid (abbreviated as O1), 2-(2-tert-butoxyethoxy) acetic acid (HO1), 5-methoxy-3-oxapentanoic acid (MO1), 5-[N-t-butyloxycarbonyl-N’-(2,2,4,6,7-pentamethyldihydro benzofuran-5-sulfonyl)]amidino-3-oxapentanoic acid (GO1), 3,6-dioxaoctanedioic acid 1-tert-butyl ester (CO1), 5-(benzyloxycarbonyl-amino)-3-oxa-pentanoic acid (O1(Cbz)), 8-(t-butyloxycarbonyl-amino)-3,6-dioxaoctanoic acid (O2), 2-(2-(2-tert-butoxyethoxy)ethoxy) acetic acid (HO2), 8-methoxy-3,6-dioxaoctanoic acid (MO2), 8-[N-t-butyloxycarbonyl-N’-(2,2,4,6,7-pentamethyldihydrobenzofuran-5-sulfonyl)]amidino-3,6-dioxaoctanoic acid (GO2), 3,6,9-trioxaundecandioic acid 1-tert-butyl ester (CO2), and 8-(benzyloxycarbonyl-amino)-3,6-dioxaoctanoic acid (O2(Cbz)).

The aim of this work is to develop a new substrate to assay the trypsin-like specificity of the human 20S proteasome that is created by DAPEG building blocks, which are N-substituted functionalized derivates of 3-diaminopropionic acid (Figure 1). The substrate preferences of the β2 subunit have been previously examined [16]. Harris and coworkers [17] described the development of new substrates selected via combinatorial library screening. The substrate peptides were labeled with 7-amino-4-carbamoylmethylcoumarin (ACC) as a reporter moiety, and a specific sequence of the β2 subunit, Ac–Glu–Ala–Nle–Arg–ACC, was used to measure the 20S proteasome activity [17]. Several substrates are currently available for measuring the trypsin-like activity of the proteasome: including commercially available 20S proteasome substrates containing C-terminal 7-amino-4-methylocoumarin (AMC), which forms an amide bond with Arg, namely Ac–Leu–Arg–Arg–AMC and Boc–Leu–Arg–Arg–AMC. Both substrates display significant selectivity for measuring trypsin-like specificity. However, they display a low specificity constant of 10^4^ M^−1^ s^−1^. Recently, Rut and co-workers applied a combinatorial chemistry approach and used positional scanning techniques to derive new selective substrates for the individual 20S proteasome subunits β1, β2, and β5. For the trypsin subunit, the most selective substrate sequence was Ac–βAla–Met(O_2_)–Thr–Arg–ACC, which displayed moderate specificity parameters (43,790 M^−1^ s^−1^). The most potent peptide in terms of specificity is the Ac–DArg–Hse–Thr–Arg–ACC (301,005 M^−1^ s^−1^) peptide, which has moderate selectivity at higher proteasome concentrations [18].

Recently [19], we established a new method for the selection and synthesis of artificial fluorogenic substrates composed of DAPEG residues (Figure 1). For the screening of 18 libraries, we created a series of compounds based on the neutrophil serine protease 4 (NSP4) substrate sequence (ABZ–X4–X3–X2–Arg–ANB–NH_2_) diversified in positions X4 to X2 with DAPEG, resulting in a total of almost 6000 compounds. The compound ABZ–Dap(O2(Cbz))–Dap(Cbz)–Dap(GO1)–Arg–ANB–NH_2_ displayed high levels of selectivity towards neutrophil-related proteases and generated a specificity constant (*k*_cat_/*K*_m_) reaching 1.3 × 10^5^ M^−1^ s^−1^. Moreover, based on putative binding, the model substrate interacted with enzymes using secondary binding sites that were distant from the active center of the protease [19].

## 2. Results and Discussion

**Figure 2:**
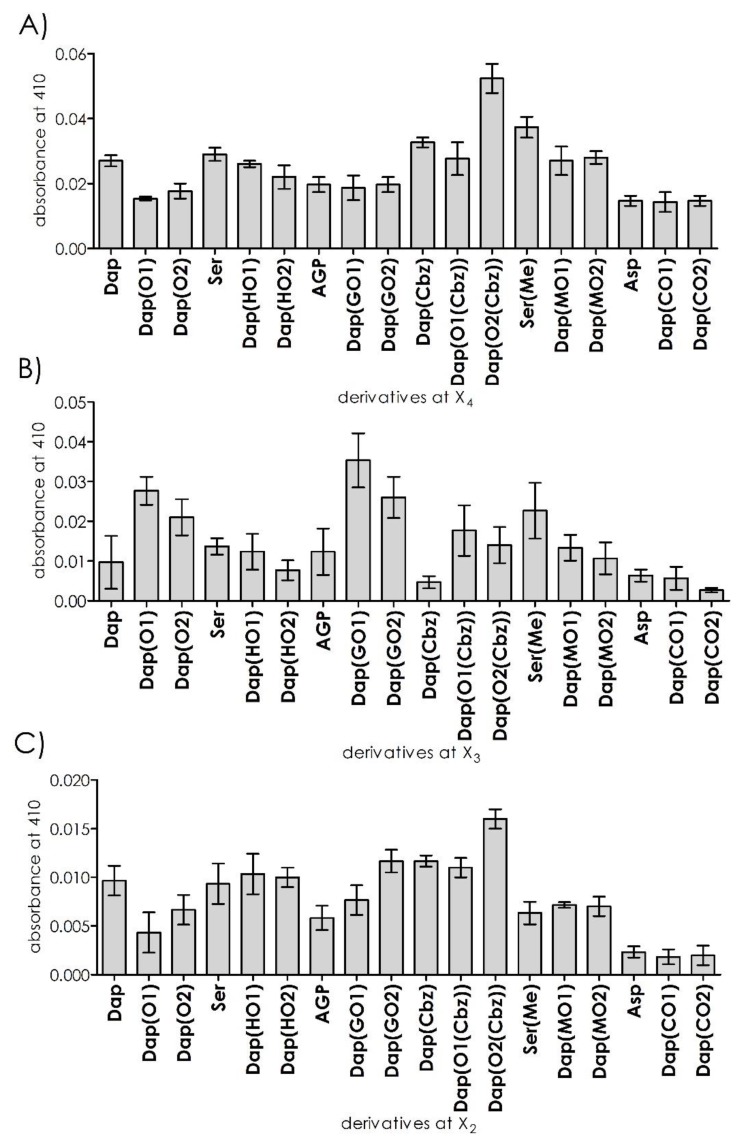
Deconvolution of the library (at an average concentration of 3.4 × 10^−6^ M) with the general formula ABZ–X4–X3–X2–Arg–ANB–NH_2_ against the 20S proteasome (1.46 × 10^−9^ M). (**A**) Cleavage rates of individual sublibraries recorded for position X4; (**B**) Cleavage rates of individual sublibraries recorded for position X3; (**C**) Cleavage rates of individual compounds modified in position X2.

Deconvolution of the synthesized library with a fixed Arg in position P1 and diversified by DAPEG residues at positions P2–P4 was performed in a solution using a mix and split approach. By analyzing the starting position (e.g., P4), we found an above-average cleavage rate for the libraries with hydrophobic groups, such as carbobenzoxy or methyl, suggesting that interaction efficacy increased with side chain length. Hydrolyzation by the 20S proteasome was significantly slower for charged or polar residues. Since the proteasome structure has three pairs of catalytically active subunits with distinct specificities (chymotrypsin, trypsin, and caspase), we repeated these experiments in the presence of a trypsin subunit inhibitor (PR671A) [20]. In effect, a 96% reduction in activity was recorded. This observation allowed us to proceed with screening at position P3 (Figure 2A).

The incubation of 18 libraries with the general sequence ABZ–Dap(O2(Cbz))–X3–X2–Arg–ANB–NH_2_ generated the highest fluorescence increases for positively charged DAPEG residues (with amino or guanidyl moieties), whereas hydrophobic or uncharged polar residues were poorly accepted at the analyzed position of P3. The highest fluorescence rate was observed for DAPEG with a guanidyl group located on its side chain, which is formed by a single ethylene unit. The velocity of Dap(GO1) was three times greater compared to that of the best P4 sub-library. The sub-library with the residue AGP (2-amino-3-guanidino-propionic acid) (shorter side chain) or GO2 was hydrolyzed significantly slower by the 20S proteasome. The same side chain length dependence was observed for DAPEG residues with amino groups on the side chain, but with a half of the rate. Repetition of the assay in the presence of the above-mentioned selective inhibitor [20] with a C-terminal vinyl sulfone warhead for the β2 subunit resulted in the total reduction of the proteolysis for this library (Figure 2B).

Next, a set of individual peptidomimetics with a defined position at P2, containing DAPEG residues or simple amino acid derivatives, were incubated with the 20S proteasome. The highest fluorescence increases were recorded for the sets of residues with aromatic groups (carbobenzoxy), followed by hydroxyl DAPEGs derivatives. In this series, the optimal side chain length increased from Dap(Cbz) and Dap(O1(Cbz)) to attain a maximum for Dap(O2(Cbz)). The same trend was observed for the hydroxy DAPEG derivatives. The basic charged side chain residues displayed moderate rates of fluorescence increases since the acidic residues significantly reduced proteolytic efficacy compared to the other tested systems. As expected, the cleavage rate increase for ABZ–Dap(O2(Cbz))–Dap(GO1)–Dap(O2(Cbz))–Arg–ANB–NH_2_ was significantly higher compared to the rates of the other tested systems. Moreover, when the assays were repeated in the presence of the selective β2 subunit inhibitor, a potent reduction in proteolysis for all systems was observed (Figure 2C).

**Figure 3:**
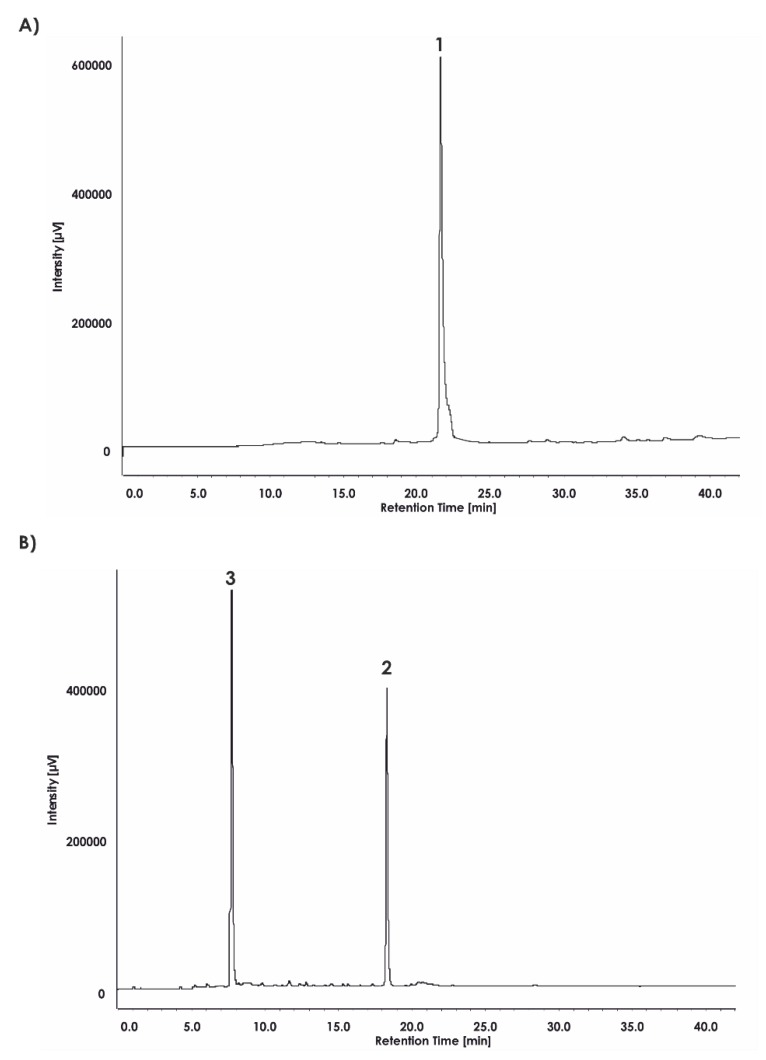
Reverse phase-high-performance liquid chromatography (RP-HPLC) analysis of (**A**) compound **1** (t_R_ = 22.41) and (**B**) compound **1** incubated with the 20S proteasome for one hour. The fragment ABZ–Dap(O2(Cbz))–Dap(GO1)–Dap(O2(Cbz))–Arg–OH (**2**) has a retention time of t_R_ = 18.2 and ANB–NH_2_ (**3**), t_R_ = 7.89.

**Figure 4:**
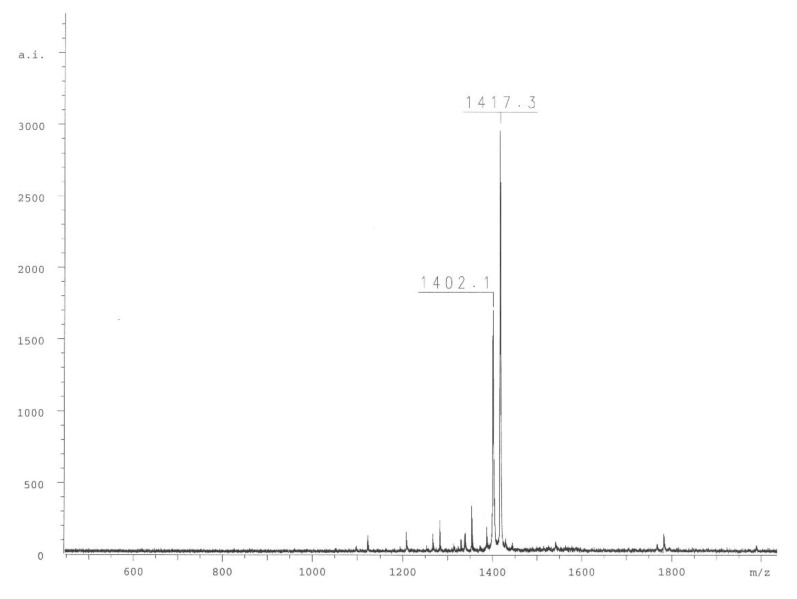
Mass spectrometry analysis of substrate **1**.

Finally, the compound ABZ–Dap(O2(Cbz))–Dap(GO1)–Dap(O2(Cbz))–Arg–ANB–NH_2_ (**1**) was analyzed. Based on its reverse phase-high-performance liquid chromatography (RP-HPLC) profile (Figure 3A and mass spectrometry analysis (Figure 4)), the spectra clearly confirmed the compound purity and its expected mass. Incubation of the final substrate with the 20S proteasome in assay buffer resulted in two products with retention times corresponding to ABZ–Dap(O2(Cbz))–Dap(GO1)–Dap(O2(Cbz))–Arg–OH (**2**) (t_R_ = 18.24) and ABZ–NH_2_ (t_R_ = 7.89) (Figure 3B).

**Figure 5:**
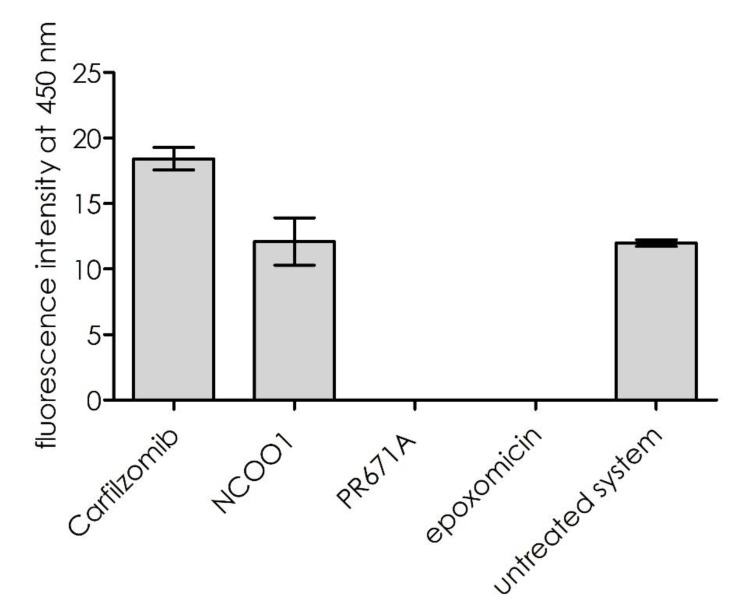
20S proteasome activity in the presence of selective proteasome inhibitors. Compounds used in the study: carfilzomib, selective inhibitor of the β5 subunit; NCOO1, selective inhibitor of the β1 subunit, PR671A, selective inhibitor of the β2 subunit; epoxomicin, general inhibitor of proteasome activity.

Cleavage was not observed in the presence of the highly selective inhibitor of the β2 sub-site (PR671A, [20]), a peptidomimetic with a C-terminal vinyl sulfone warhead. As shown in Figure 5, inhibition of the trypsin-like sub-site (using the general proteasome inhibitor, epoxomicin, or the PR671A inhibitor described above) resulted in the proteolytic suppression of substrate **1**. Inhibition of the remaining proteolytic sites, β5 and β1, did not influence this process. However, for carfilzomib, a slight activation of the cleavage rate was observed. This result indicates that the substrate was hydrolyzed exclusively by the β2 sub-site.

**Figure 6:**
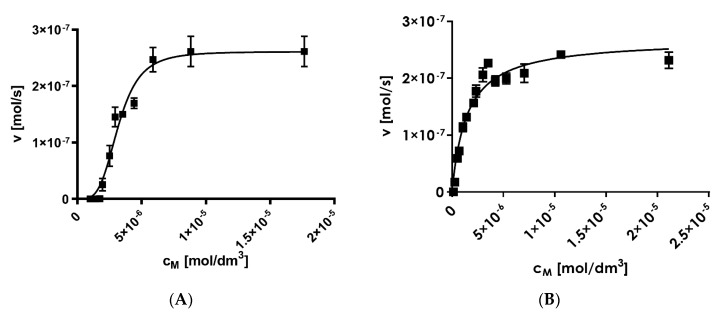
Kinetic curves for the 20S proteasome in two systems: (**A**) substrate alone and (**B**) in the presence of the artificial activator, 0.01% sodium dodecyl sulfate (SDS).

Fluorescent substrate cleavage followed sigmoidal kinetics, which is characteristic for allosteric enzymes, with *K*_m_ = 3.22 ± 0.02 μM, *k*_cat_ = 245 ± 21 s^−1^, and *k*_cat_/*K*_m_ = 7.61 × 10^7^ M^−1^ s^−1^ (Figure 6A). For the same experiments performed in the presence of the artificial proteasome activator, sodium dodecyl sulfate (SDS), the *K*_m_ values were twice as large (*K*_m_ = 6.84 ± 0.12 μM), but the turnover value remained at a comparable level (*k*_cat_ = 221 ± 18 s^−1^ and *k*_cat_/*K*_m_ = 3.23 × 10^7^ M^−1^ s^−1^). The obtained kinetic values for substrate **1** were much greater than those reported for the other substrates of the proteasome reported by other groups (Table 1). Moreover, the β2 subunit activity of the 20S proteasome was not significantly altered in the presence of the 20S proteasome activator, SDS (Figure 6B).

To elucidate the data from the deconvolution experiments, we performed in silico docking using the PLANTS program [23]. For calculations, we used the crystal structure of the human 20S proteasome (protein database (pdb) code 4R67) in complex with the carfilzomib potent inhibitor of the proteasome [24]. β2 and β3 (using the yeast nomenclature) were extracted and used for the docking simulations. Two sequential docking calculations were performed under the following conditions: The number of ants used by the software to search for the protein binding pocket was set to 40, and the number of resulting conformations was set to 30.

In the first “blind docking” experiment, only the space around the S1 and S3 binding pockets was declared. In the second docking experiment, the positional restraint was set on the Arg residue located in the S1 binding pocket. Using this procedure, we sought to verify that the Arg residue was in the vicinity of the S1 binding pocket. Our analysis revealed that the only residue located in the binding pocket was Arg. The second docking sequence was performed with the distance restraint set to the Arg residue. For this subunit, the S1 binding pocket was considerably larger than the β1 and β5 subunits and was comprised of the following residues: His35, Thr52, and Asp53. Analysis of the binding interface of carfilzomib with β2 and β3 showed that the Leu side chain (P1) was in the S1 binding pocket. In our case, this was the residue in position X3–Dap(GO1). According to the deconvolution experiments, four residues were optimal for this position: Dap(GO1), Dap(GO2), Dap(O1), and Dap(O2). Since the S1 binding pocket was acidic, basic residues were ideal for creating an optimal interaction. The residues Dap(CO1), Dap(CO2), and Asp did not work at all, whereas the Ser and Dap residues were too small to accommodate the binding pocket. The other possibility was to create π–π interactions with the ligand by using His at position 35. However, the S1 binding pocket was too small to accommodate the three stacking rings Dap(Cbz), Dap(O1(Cbz)), and Dap(O2(Cbz)).

The residue at position X2 was located in the S3 binding pocket. Analysis of the binding interface of carfilzomib with β2 and β3 showed that the Leu side chain (P3) was located in the S3 binding pocket. Asp125 was located at the entrance of the S3 binding pocket. Therefore, all acidic residues may have had a negative influence on the binding affinities. This was confirmed by the deconvolution experiments, since residues Asp, Dap(CO1), and Dap(CO2) did not bind at all. The S3 binding pocket passed through the interface between the β2 and β3 subunits. Only the Dap(O2(Cbz)) residue was long enough to accommodate the entire space of the binding pocket.

Most of the residues at position X4 worked well since they were in the grove of the proteasome. The greater the number of interactions, the greater the binding affinity, which was true for Dap(O2(Cbz)).

**Figure 7:**
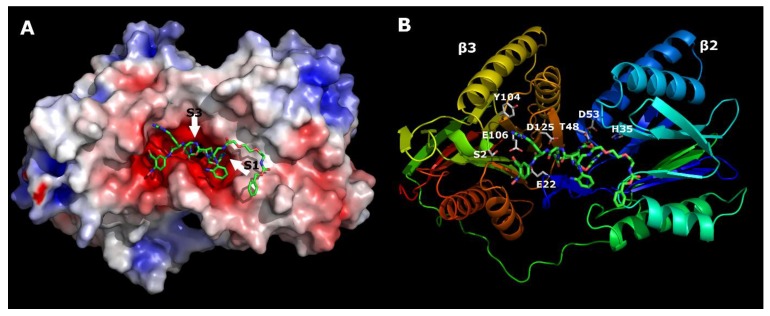
Theoretical model of the ligand ABZ–Dap(O2(Cbz))–Dap(GO1)–Dap(O2(Cbz))–Arg–ANB–NH_2_ interacting with β2 and β3 subunits of the 20S proteasome. (**A**) Electrostatic surface (red: negative charge; blue: positive charge), (**B**) the most important residues at the proteasome–ligand interface. Arrows indicate the location of the S1 and S3 binding pockets.

According to Reference [23], for trypsin-like sites, the S1 and S3 binding pockets are larger than those for caspase- and chymotrypsin-like sites. This was clearly visible from our 20S proteasome structure analysis. Presumably, the structure of the mentioned binding pockets explains the selectivity of our ligand. According to the molecular modeling results, two relatively large residues were located in these pockets (Figure 7). This was not possible for caspase- and chymotrypsin-like sites. Similarly, these sites did not have acidic environments like those of trypsin-like sites. Only this site had perfect conditions for the base-like residues Dap(GO1) or Dap(GO2).

**Figure 8:**
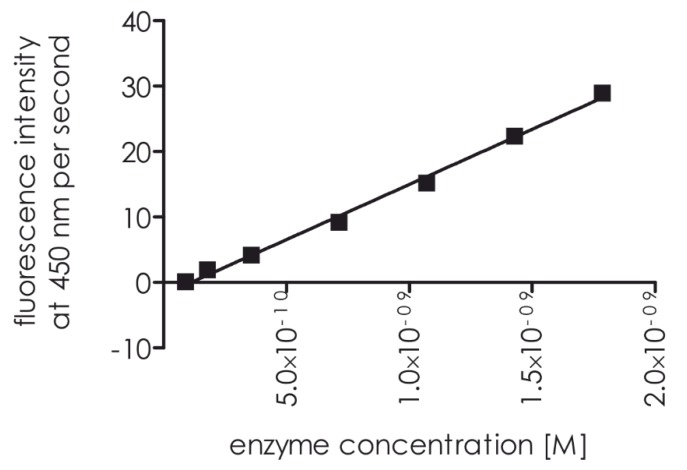
The 20S proteasome assay detection limits. The following 20S proteasome concentrations were analyzed: (1) 2 × 10^−9^ M, (2) 1.5 ×10^−9^ M, (3) 1 × 10^−9^ M, (4) 0.75 × 10^−9^ M, (5) 0.4 × 10^−9^ M, (6) 0.1 × 10^−9^ M, and (7) 0.05 × 10^−9^ M.

The titration of decreasing proteasome 20S concentrations with substrate **1** resulted in a concentration-dependent increase in fluorescence to 8.93 10^−11^ M (Figure 8).

**Figure 9:**
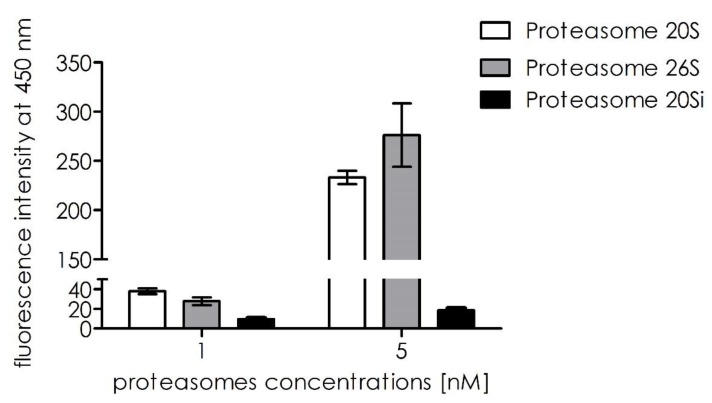
Substrate cleavage rates for the 20S, 26S, and 20S immunoproteasome entities at equimolar concentrations.

Next, we tested how substrate **1** was processed by the other proteasomal entities, such as proteasome 26S and immunoproteasome 20S. Initial screening showed that substrate **1** was selectively proteolyzed by the 20S proteasome (Figure 9).

**Figure 10:**
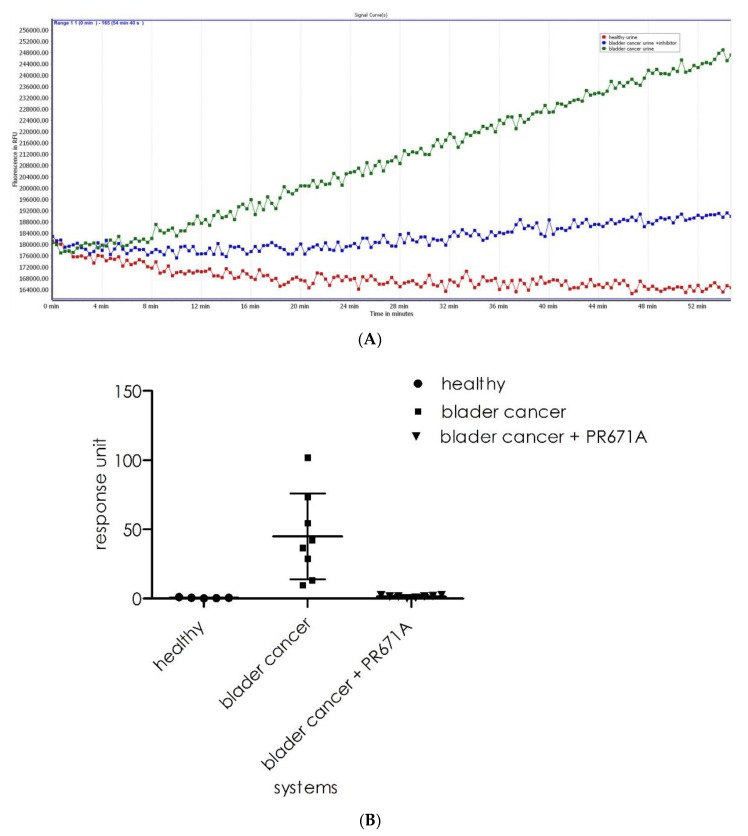
(**A**) Fluorescence curves for the three the systems: healthy urine (red line), bladder cancer urine (green line), and inhibitor-treated bladder cancer urine (blue), (**B**) aggregate analysis of the healthy and bladder cancer urine and inhibitor-treated urine.

**Figure 11:**
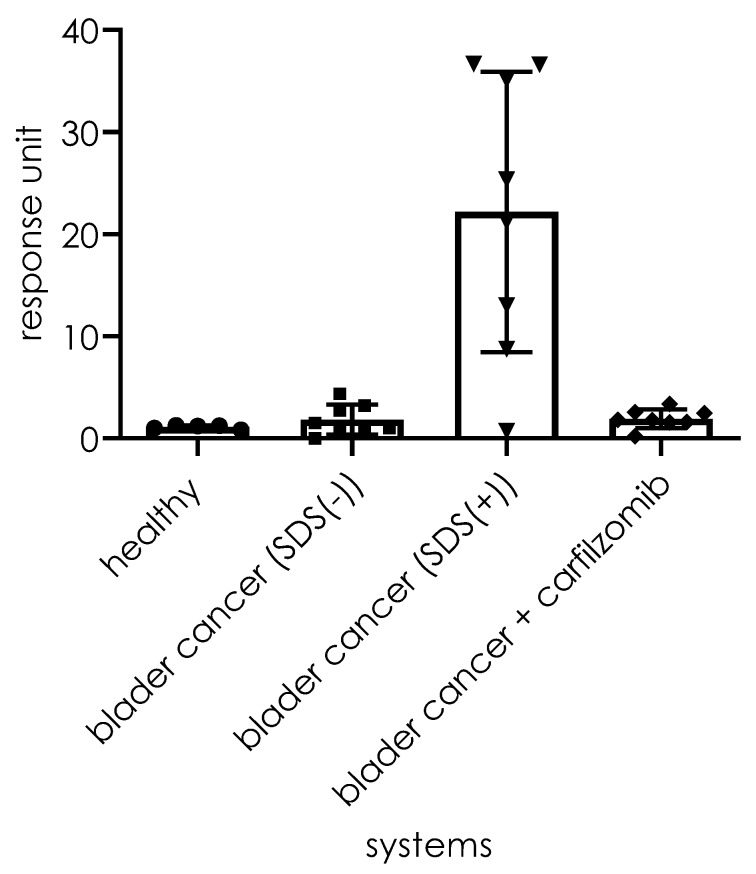
Aggregate analysis of the healthy and bladder cancer urine and inhibitor-treated urine.

Next, we evaluated the ability of substrate **1** to detect proteasome activity in biological samples. As previously reported, elevated 20S proteasome activity in human urine is often linked to a diagnosis of bladder cancer [21]. To verify whether substrate **1** could be used in such an assay, we recorded its cleavage intensity in healthy (*n* = 5) urine samples and bladder cancer urine (*n* = 8) samples. As observed (Figure 10A), the healthy urine samples displayed insignificant background levels of fluorescence (red line). However, for the urine sample from bladder cancer patients, the proteasome activity resulted in a fluorescence increase that was observed in all samples (green line). The incubation of samples with a specific 20S proteasome inhibitor resulted in significant reductions in cleavage rates (blue line). As shown in Figure 10B (a summary of the experiments), there is a significant difference in the activity recorded for the healthy (*n* = 5, mean 0.1 ± 0.04) and bladder cancer urine samples (*n* = 8, mean 48.4 ± 28.1). The results for the analysis of the same samples with the substrate design for the chymotrypsin subunit (β5) of the 20S proteasome were acquired under the same conditions used for substrate **1** and for the system supplemented by the artificial activator (SDS) of the 20S proteasome, as presented in Figure 11. For systems lacking SDS, the fluorescence increase is visible for only 2 out of the 8 bladder cancer samples. An increase in fluorescence was observed for seven systems (1–5, 7, and 8) with SDS among the assay systems. For one sample (N° 6), the rate was insignificant. For all systems, the samples that originated from healthy persons displayed no visible fluorescence increase.

## 3. Materials and Methods

Synthesis of the ANB-based library, where ANB is 5-amino-2-nitrobenzoic acid, was initiated by the deprotection of the amino groups of Tenta Gel SRAM resin (Rapp Polymeres, Tubingen, Germany) with 20% piperidine in dimethylformamide (DMF). The attachment of 5-amino-2-nitrobenzoic acid (ANB) used the following reagents: *N*,*N*,*N*′,*N*′-tetramethyl-O-(benzotriazol-1-yl)uronium tetrafluoroborate (TBTU)/4-dimethylaminopyridine (DMAP). After this procedure, the resin was mixed with 5% *N*-methylmorpholine in DMF, and the solution was removed. This step was repeated twice. Next, three equivalents of ANB were dissolved in DMF, and three equivalents of TBTU were added, followed by two equivalents of DMAP. This solution was added to the resin, and after 30 s, six equivalents of *N*,*N*-diisopropylethylamine (DIPEA) were added. The mixture was then stirred for 3 h.

The solution was filtered using a Schott funnel (MO5), and the resin was washed in DMF. This procedure was repeated three times. Next, the first amino acid residue was coupled using a previously described method [21,25]. A nine-fold amino acid excess was applied to the active resin sites as follows: the amino acid residue was dispersed in pyridine (for each 1 g of peptidyl resin, 10 mL pieridine was used). The solution was stirred until its temperature reached −15 °C, and then nine equivalents ofphosphorus oxychloride POCl_3_ were added. The mixture was stirred for 20 min at −15 °C, for 20 min at room temperature, and then for 6 h in an oil bath at 40 °C. After deprotection of the Fmoc-protecting group with 20% piperidine in DMF, the peptide chain was elongated as follows: The solid support was split into 18 identical portions, with six of them containing one of the following standard protected amino acid derivatives: Fmoc–Asp(tBu), Fmoc–Ser(OtBu), Fmoc–Ser(OMe), Fmoc–Dap(Cbz), Fmoc–Agp(Boc)_2_, and Fmoc–Dap(Boc), which were attached using a standard Fmoc synthesis procedure. Fmoc–Dap(Mtt) was introduced to the remaining 12 portions, and 4-methyltrityl (Mtt) was removed using a previously described procedure [19] (1% TFA in DCM with the addition of 2% triisopropylsilane). This mixture was added to all 12 portions and stirred for 15 min. Complete Mtt removal was verified by adding a minimal portion of peptidyl resin with pure TFA. A yellow color was used to monitor the absence of free Mtt groups. This procedure was repeated until no absorbance increases at 410 nm were recorded. This step was followed by adding 1% DIPEA in DMF solution to each system for 3 × 1 min, resulting in 12 portions of a Fmoc–Dap–Arg–ANB–polymer. To each resin aliquot, mono-protected heterobifunctional polyethylene glycol moieties (PEG: O1, HO1, MO1, GO1, CO1, O1(Cbz), O2, HO2, MO2, GO2, CO2, O2(Cbz)) were introduced while applying equimolar quantities of PEG/DIPCI/HOBt in a DMF/NMP (1:1 (v/v)) solution. Subsequently, the following fully protected PEG derivatives were applied: 5-(t-butyloxycarbonyl-amino)-3-oxapentanoic acid (O1), 8-(t-butyloxycarbonyl-amino)-3,6-dioxaoctanoic acid (O2), 5-[N-t-butyloxycarbonyl-N’-(2,2,4,6,7-pentamethyldihydrobenzofuran-5-sulfonyl)]amidino-3-oxapentanoic acid (GO1), 8-[N-t-butyloxycarbonyl-N’-(2,2,4,6,7-pentamethyldihydrobenzofuran-5-sulfonyl)]amidino-3,6-dioxaoctanoic acid (GO2), 5-(benzyloxycarbonyl-amino)-3-oxa-pentanoic acid (O1(Cbz)), 8-(benzyloxycarbonyl-amino)-3,6-dioxaoctanoic acid (O2(Cbz)), 2-(2-tert-butoxyethoxy) acetic acid (HO1), 2-(2-(2-tert-butoxyethoxy)ethoxy) acetic acid (HO2), 3,6-dioxaoctanedioic acid 1-tert-butyl ester (CO1), 3,6,9-trioxaundecandioic acid 1-tert-butyl ester (CO2), 5-methoxy-3-oxapentanoic acid (MO1), and 8-methoxy-3,6-dioxaoctanoic acid (MO2). Coupling completeness was monitored using a Kaiser test. A lack of free amino groups facilitated the next step in library synthesis, where all resin portions were mixed and divided into 18 parts. A total of 20% of the peptidyl resin from each portion was removed and stored for further deconvolution processes. The above-mentioned method was repeated once again. Next, the tert-butyloxycarbonyl derivative of 2-amino benzoic acid (Boc–ABZ–OH) was used and coupled to the N-terminal amino group via the procedure used earlier.

After completing the synthesis, sub-libraries or particular compounds were detached from the solid support using a TFA/phenol/triisopropylsilane/H_2_O mixture (88:5:2:5 (v/v)) [25]. Compound purity and synthesis were verified using an RP-HPLC ChromNAV (Jasco, Japan) with a Kromasil 100 C8 column (Knauer, Germany) coupled to a UV–Vis and fluorescence detector. A linear gradient was used from 10% to 90% solution B over 45 min (A: 0.1% TFA; B: 80% acetonitrile in A). The peptides were assayed at 208 nm. The molecular weights of the obtained compounds were obtained by mass spectrometry and recorded on a Biflex III MALDI TOF mass spectrometer (Bruker Daltonics, Hamburg, Germany) using a matrix (α-cyano-4-hydroxycinnamic acid).

### 3.1. Preparation of Peptide Libraries

The peptide libraries were synthesized using a portioning-mixing method [26]. Initially, 17.7 g of solid support (Tenta Gel SRAM Tubingen, Germany) was used (i.e., ABZ–X4–X3–X2–Arg–ANB–NH_2_). A double excess of each library building block (amino acid or monoprotected PEG derivative) was used for the synthesis. Other techniques applied in the library preparation are described above. After attachment of the ANB followed by Fmoc–Arg(Pbf) and Fmoc removal, the initial amount of the resin was split into 18 equal parts (related to the number of building blocks used for synthesis of the library). After successful attachment of the chemical derivatives, 15% of the resin portion with ANB–Arg(Pbf)–P2 was put aside until the deconvolution process. The Fmoc protection group was removed from the remaining 85% of the library, and the total amount of the resin was again split into 18 equal parts. This synthetic procedure, referred to as “mix and split”, was repeated until position P4 was reached. In this final stage of library construction, the resin was not mixed, but all peptidomimetics were detached from the resin and subjected to enzymatic studies performed by applying the iterative method. In the first stage, the most active residue was selected via an enzymatic assay and, in subsequent synthetic steps, this residue was attached to the remaining 15% portion of the resin. This process was repeated (three times) until the final substrate sequence was selected [27].

### 3.2. Enzymatic Studies

For enzymatic studies, the human 20S proteasome, 26S proteasome, and 20S immunoproteasome were purchased from Boston Biochem (Cambridge, MA, USA). The 20S proteasome concentration for library deconvolution was in the nanomolar range, and this proteasome entity was used for the main study. Deconvolution of the peptide libraries was performed using the iterative solution method [28]. Dimethyl sulfoxide (DMSO) was used as a solvent for preparation of the stock solution (5 mg/mL) for each lyophilized sub-library. This stock solution was diluted ten times using an assay buffer (50 mM Tris/HCl buffer (pH 8.2)) for the 20S and 20Si proteasome, and for 26S, the buffer was supplemented with 40 mM KCl, 2 mM EDTA, 1 mM DTT, 100 µM ATP, and 50 µg/mL bovine serum albumin (BSA). The volume composition of each well of the 96-well plate was: 20 µL library, 160 µL assay buffer, and 20 µL 20S proteasome solution at a concentration of 1.07 × 10^−9^ M. Absorbance or fluorescence measurements were performed on a FluoroStar OMEGA multimode microplate reader (BMG, Ortenberg, Germany). Absorbance increases were monitored at 410 nm. The excitation and emission wavelengths were 320 and 450 nm respectively, for the ABZ/ANB. Enzymatic hydrolysis of the peptide was performed in the assay buffer at 37 °C and continued over 60 min.

### 3.3. Determination of Kinetic Parameters

The procedures to determine the kinetic constants *K*_m_ and *k*_cat_ are described below. Increasing concentrations of substrate **1** ranged from 1 × 10^−6^ to 2 × 10^−5^ M. In parallel, a kinetic assay with the artificial activator SDS at 0.01% was also performed. The data were fitted to an allosteric plot using GraphPad prism v6.0 (GraphPad Software Inc., La Jolla, CA, USA). The specificity constants (*k*_cat_/ *K*_m_) were calculated from the *k*_cat_ and *K*_m_ values. Measurements were performed using a 20S proteasome concentration of 1.07 × 10^−9^ M. Three to five measurements were performed for each compound dilution (the systematic error, defined as the standard deviation, never surpassed 20%). The initial hydrolysis rates were used as the amount of enzymatic activity for the tested compounds. All kinetic study details and kinetic parameters have been described elsewhere [21,25].

### 3.4. Determination of Proteolytic Cleavage Patterns

For each sample of the diluted substrate (concentration; 3.1 × 10^−6^ M), an appropriate amount of proteasome (1.07 × 10^−9^ M) was added, and the solution was incubated for 5, 30, and 60 min. The proteolytic reaction was monitored by RP-HPLC.

### 3.5. Titration

A total of 160 µL assay buffer was added to each well of the 96-well plate, containing a constant substrate concentration (1.34 × 10^−5^ M) (20 µL), followed by 20 µL of the 20S proteasome, with concentrations ranging from 0.05 to 2 nM. In total, seven different concentrations were used. The excitation and emission wavelengths were 320 and 450 nm, respectively. Enzymatic hydrolysis of the peptide was performed in the previously described buffer at 37 °C and continued over 30 min. A linear fragment of the curve was chosen, and the slope was calculated for each system. All measurements were performed in triplicate.

### 3.6. Inhibitory Studies

A black, 96-well flat-bottomed microplate (Brand GMBH, Wertheim, Germany) was used for the inhibitory studies. Two inhibitors, carfilzomib (a selective inhibitor of the β5 subunit of the 20S proteasome) and epoxomicin, were purchased from Merck (Darmstadt, Germany) and used at final concentrations of 1.15 × 10^−6^ M and 5.0 × 10^−5^ M, respectively. The β2 inhibitor, PR671A [20], and the β1 inhibitor, NCOO1 [21], were used at a concentration of 5 × 10^−6^ M. These reagents were kindly provided by Alexei Kisselev from Dartmouth University (Dartmouth, NH, USA). The concentration of 20S proteasome was 1.07 × 10^−9^ M. The buffered proteasome solution was mixed with 10 µL inhibitor solution. This mixture was incubated for 30 min at 37 °C. After this period, the substrate solution was added at a concentration of 1.34 × 10^−5^ M, and the fluorescence was measured over time at 320 nm excitation and 450 nm emission wavelengths. All measurements were performed in triplicate.

### 3.7. Biological Studies

All urine samples were purchased from St. Vincent de Paul Hospital in Gdynia, Poland (contract no. JN/777/R720/08.11.17). All methods and procedures were performed in accordance with this contract agreement. Participants were informed about the study and sample collection, and their written consent was collected. Urine samples did not reveal any personal information except for gender, age, and disease stage. Therefore, the samples were considered unnamed. The samples of human urine were taken during mid-urination and kept for 48 h at −80 °C until required. All samples used for assay development were taken from healthy volunteers (*n* = 5), and oncological samples were obtained from patients diagnosed with bladder cancer (*n* = 8).

The urine samples were thawed at room temperature, gently vortexed, and briefly centrifuged (<20 s) to collect the sample at the bottom of the tube. A total of 80 μL urine was transferred to a 96-well microplate and mixed with assay buffer and substrate **1**. Proteasome activity was assayed as described earlier. Briefly, 80 µL of urine from healthy volunteers, urine from patients diagnosed with bladder cancer, and bladder cancer urine supplemented by the inhibitor PR671A were mixed with 200 µL of the assayed buffer and 20 µL of the substrate at a concentration of 1.34 × 10^−5^ M, and the fluorescence was quantified. An excitation wavelength equal to 320 nm and an emission wavelength of 450 nm were used.

In parallel, the urine samples were analyzed using the previously developed substrate ABZ–Val–Val–Ser–Tyr–Ala–Met–Gly–Tyr(3-NO_2_)–NH_2_ [21] in two systems: one with the artificial activator SDS at 0.01% and the second without any SDS. The fluorescence of the system was recorded over time. The same conditions for excitation and emission were used as above. All measurements were performed in triplicate.

The resulting data were analyzed using Graphpad Prism version 6.0 software (GraphPad Software Inc., La Jolla, CA, USA). All fluorescent measurements were performed in triplicate and represented by the mean ± standard deviation (SD). A two-tailed Mann–Whitney test was applied for the statistical analysis (*p* < 0.0001).

## 4. Conclusions

We developed a new 20S proteasome fluorescent peptidomimetic probe with superior kinetic parameters, yielding 7.61 × 10^7^ M^−1^ s^−1^. The synthesized substrate was cleaved at a minimal 20S proteasome level at 10^−11^ M. Based on a putative model derived from molecular docking, the probe interacts with the 20S proteasome using secondary binding sites located distally from the catalytic Thr of the β2 subunit. Moreover, this newly developed substrate is, to our knowledge, one of the best substrates designed for the β2 subunit of the 20S proteasome. The majority of substrates in the proteasome assay required the presence of SDS as an artificial activator in the system. Substrate **1** could be used without such an addition, making the assay much simpler to perform. Using our substrate, we detected 20S proteasome activity in the human urine samples from bladder cancer patients. This observation could be useful for the noninvasive diagnosis of this severe disease.

## Figures and Tables

**Table 1:** Overview of the fluorogenic substrates of the trypsin-like subunit of the 20S proteasome.

No.	Sequence	*K_m_* (µM)	*k_cat_* (s^−1^)	*k_cat_/K_m_* (M^−1^ s^−1^)	Ref.
**1**	ABZ–Val–Val–Ser–GNF–Ala–Met–Gly–Tyr(3-NO_2_)–NH_2_	4.24 ± 0.87	8.41 ± 0.57	1,983,100 ± 2754	[21]
**2**	Boc–Leu–Arg–Arg–AMC			<500	[22]
**3**	Ac–DArg–hSer–Thr–Arg–ACC	9.43 ± 0.82	2.84 ± 0.22	301,005 ± 28,471	[18]
**4**	Ac–βAla–Met(O)_2_–Thr–Arg–ACC	50.9 ± 2.99	2.23 ± 0.07	43,790 ± 3233	[18]

ABZ = 2-aminobenzoic acid, GNF = 4-guanidine-L-phenylalanine, Tyr(3-NO_2_) = 3-nitro-L-tyrosine, AMC = 7-amino-4-methylcoumarin, ACC = 4-carbamoylmethylcoumarin, βAla = beta-alanine, Ac = acetyl moiety, and Met(O)_2_ = methionine sulfone.
